# Crisis Information Management: From Technological Potential to Societal Impact

**DOI:** 10.1007/978-3-030-65355-2_22

**Published:** 2021-03-20

**Authors:** Kenny Meesters

**Affiliations:** 1 Tilburg University, Tilburg, The Netherlands; 2 Tilburg University, Tilburg, The Netherlands; 3 Tilburg University, Tilburg, The Netherlands; 4 Tilburg University, Tilburg, The Netherlands; Department of Management, Tilburg School of Economics and Management, Tilburg, The Netherlands

## Abstract

Every day we are making decisions, both in our personal and professional lives. These decisions range from choices regarding our mode of transport to our daily grocery shopping, and from investment decisions to choices about marketing strategies. Today, for each of these decisions, we can rely on a wide range of information sources and tools to aid us in these decision-making processes. Spurred on by technical developments and economic incentives, information has become a common commodity in our society.

When confronted with a crisis, we find ourselves in an unexpected and unknown situation in which quick action is needed to remedy the situation or prevent escalation. In such cases, information plays a vital role, for example, in assessing our options and reducing uncertainty. Information allows decision makers to assess the situation, evaluate alternatives, and coordinate efforts between different stakeholders, for example. The phrase “information saves lives,” commonly uttered in crisis responses, exemplifies this importance. Neverheless, existing approaches to getting information may no longer be sufficient, reliable, or even accessible. A crisis therefore requires all stakeholders, from formal responders to affected citzens, to quickly re-design their information flows using an effective organization of people, technology, processes, and sources.

Every day, we make decisions, both in our personal and professional lives. Ranging from choices regarding our mode of transport to our daily grocery shopping, and from investment decisions to choices about marketing strategies. Today, we can rely on a wide range of information sources to aid us in these decision-making processes. Mobile phones, online communities, and a host of digital services give access to a wide range of information. In short, spurred on by technical developments and economic incentives, information has become a common commodity in our society (Lissenden et al. 2015).

When faced with a crisis situation, we are confronted with an unexpected and unknown situation in which quick action is needed to remedy the situation or prevent escalation to worse. For example, in these situations, scarce resources and capacities have to be allocated while the knowledge about the situation is often limited. This time pressure to act, the high level of uncertainty, and the ambiguity of actions to undertake make crisis management specifically challenging (van den Homberg et al. 2014). Information plays an important role in reducing uncertainty in a crisis. Information allows decision makers, for example, to assess the situation, evaluate alternatives, and coordinate efforts between different stakeholders. The phrase “information saves lives,” commonly uttered in crisis responses, exemplifies this importance (Comfort et al. 2004).

Therefore, the challenge during a crisis situation is to obtain relevant, accurate, and timely information to support key stakeholders in their decision-making process (Gralla et al. 2015). While existing information is often rapidly outdated or inaccurate in these situations, the underlying information technologies enable various ways to quickly obtain information. In fact, today, there is a myriad of possibilities to manage the information available to emergency responders, information managers, and decision makers. Social media and crowd-sourcing techniques can prove a valuable source of information to understand the key issues faced; data analytics and artificial intelligence can be used to uncover trends and key events in a crisis; web platforms and repositories are used to disseminate information products (Meier 2011).

However, there is an important distinction to be made between available information and actionable information (Derczynski et al. 2018). The information, or rather possible information, generated by these tools and available to decisions makers can in fact be extensive. To utilize the potential of all this information and its supporting technologies, a better alignment is needed between information availability spurred on by new technologies and its effective use in the decision-making process. Therefore, today, the challenge is no longer the availability of the information but rather designing, structuring, and managing flows of information to support the decision-making process in a networked manner (Coyle and Meier 2009).

## COVID-19 and Information Management

During the COVID-19 outbreak and the response to this outbreak, the importance of information to support decision makers became abundantly clear. In the Netherlands, public health care agencies tracked the number of infected people, hospitals reported the capacity on a daily basis, and medical suppliers kept close track of the stocks. As the outbreak continued, the stream of information continued to grow: transport agencies reported the number of travelers, information from telecommunication services were used to track crowd movements, and financial services began reporting the economic impacts. Across the globe, similar efforts were undertaken to collect, process, and disseminate information. The United Nations Office for Coordination of Humanitarian Affairs (UN OCHA), for example, set up dedicated sections at their Humanitarian Data Exchange to support global monitoring, and John Hopkins University launched their Coronavirus Resource Center.

A complicating factor in this specific crisis for decision makers is the widespread and long-lasting impact of the pandemic across all facets of our societies. This also meant that the number of actors involved grew substantially, and with the addition of each actor, the information network expanded. Each actor was generating and disseminating his/her own information but also collecting information through their networks to fulfill their information needs. At the same time, an increasing number of initiatives that aim to support the information management tasks were developed and offered. These included analytics to discern trends, models to assess the effects for different interventions, or systems to monitor the situation. These developments provided decision makers and crisis response organizations with a slew of options to choose from. In a short time, the complexity of managing information grew exceptionally fast.

Managing information in general, and specifically the flow of information, has become crucial in the COVID-19 outbreak. More than obtaining the information itself, the purposeful design of information management processes and systems has become crucial (Meissner et al. 2002).

## From Potential to Impact

The COVID-19 outbreak presented new challenges to emergency responders. The multifaceted impact of the outbreak on our society over a longer period significantly increased the number of actors, decision makers, information needs, and thus information management tasks. The technical building blocks for systems to support these tasks are available, ranging from technologies for obtaining data automatically and in large quantities to systems that support processing this data into information and visualizing the results.

While technologies presented opportunities to gain access to the increased volume of information, process the information to form key insights, and enable organizations to exchange this information, the nature of the pandemic outbreak warrants a reconsideration of the information management approaches commonly used. The duration of this crisis, technological advancements, and the abundance of information facilitate and even necessitate the need for a more structural approach to information management. The challenge is no longer technical in nature or due to the absence of information but rather the effectively management of this potential to support decision makers. This is requiring organizations to consider not only the technical and information side but also the organizational and human aspects of their organizations.

**Fig. 22.1:**
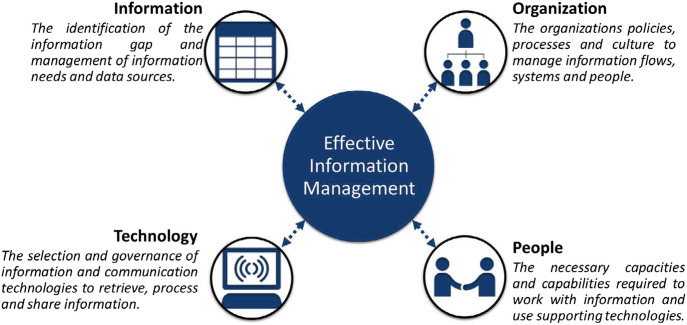
Elements for Effective Information Management (source: author)

As illustrated in Fig. 22.1, leveraging the potential of information and technologies is not only a technical challenge but it also requires organizations to reconsider their procedures, capacities, and culture. It requires alignment between the organizational aspects and human factors. Specifically, regarding the COVID-19 crisis, two specific aspects are of importance due to the increased size of the information landscape:

- **Information Needs**: To manage information flows effectively, it is important to understand the information needs. Identifying the needs of the decision makers provides guidance in assessing the value of incoming information, the required categories of information, and the required quality. More importantly, determining and monitoring the information needs also enables organizations to determine if there is any information missing and to actively fill these gaps, rather than act on the available information. Using this “gap” analysis, additional sources can be identified or developed, or further information processing can be included to align the information with the decision maker’s needs (Meesters and Van de Walle 2013).
- **Organization and People**: To effectively manage information and use the available technologies, the fit with the organizational processes and capacities is a key consideration. There are numerous options available to emergency responders and organization. However, selecting, effectively using, and integrating these options requires specific knowledge and capacities. It also requires organizations to adapt a different approach towards a more networked information management approach. Collaborations can be formed not only for the exchange of information but also for the required capabilities to manage it (Paulus et al. 2018).

## Information as Aid

The above developments and trends illustrate important developments and considerations for emergency responders and crisis management organizations to effectively leverage the potential of information during a crisis situation. However, decision-making during crisis information, and subsequently the need for information, does not only pertain to crisis responders, government agencies, or organizations with a public role. As in every emergency, civil society plays a critical role in the crisis response (Walter 2004). Every organization, community, and individual is making choices in these uncertain times. In fact, individual decisions made by citizens largely determine the effectiveness of the crisis response. This individual responsibility is even more important during pandemics and other public health emergencies.

It could be argued that today, and especially during the COVID-19 outbreak, information itself has become a primary need. Moreover, through technologies such as web 2.0, more and more people are actively creating and sharing digital information. Social media, for example, allow people to share first-hand experiences directly with a large community (Coyle and Meier 2009). This warrants an important change for crisis responders, government agencies, and our society in general, and requires adapting the information management principles used in crisis response organizations (Meesters et al. 2019).

Notwithstanding this potential, there are important considerations to be made in light of these developments. Especially in an emergency, those who are more vulnerable will likely have less access to critical information. The reduced online presence, in general, will also result in underrepresentation in digital sources. In contrast, those with information literacy skills, access to information technologies, and strong networks will not only have access to a larger amount of information to base their decision-making on but can also leverage their connections and social networks to obtain crucial support. The COVID-19 outbreak, in particular, has shown the importance of the ability to “stay connected” by digital means. During these crisis situations when people are most vulnerable, the digital divide increases even further (Comes et al. 2019).

## Enabling the New Common Through Information

As we move to a new common, decisions continue to be made by a wide range of actors, not in the least the our communities and their individual members. The effective transition to a new common in our society depends on the alignment and ability to make informed decisions. Therefore, we need to expand the information management landscape of crisis management. This requires the organization to reexamine their information management approach. Not only in terms of technologies employed, such as social media, and consideration of the information needs of the community members. But specifically in relation to their organizational capabilities, policies, and processes.

While careful ethical, societal, and security aspects have to be considered, the need for and value of information in the decision-making process reaches beyond the scope of emergency responders and their organizations. Through the exchange of information, communities and civic organizations can not only be informed but even empowered to effectively participate in both the response and information management processes (Piccolo et al. 2017). They can deliver valuable information to decision-makers, and vice versa their own decisions can be aligned with other stakeholders as well (Meesters et al. 2021). However, to leverage this potential, a paradigm shift is required in crisis organizations and their information management towards an inclusive and reciprocal approach.
